# Predictive validity of developmental milestones for detecting limited intellectual functioning

**DOI:** 10.1371/journal.pone.0214475

**Published:** 2019-03-28

**Authors:** Eline Vlasblom, Magda M. Boere-Boonekamp, Esther Hafkamp-de Groen, Elise Dusseldorp, Paula van Dommelen, Paul H. Verkerk

**Affiliations:** 1 Department of Child Health, TNO, Leiden, The Netherlands; 2 Department of Health Technology and Services Research, Technical Medical Centre, University of Twente, Enschede, The Netherlands; 3 Rivas Zorggroep, Gorinchem, The Netherlands; 4 Institute of Psychology, Leiden University, Leiden, The Netherlands; University of Copenhagen, DENMARK

## Abstract

Developmental milestones are commonly used in child health care, although from many milestones the predictive validity has not been adequately assessed. We aimed to determine the predictive validity of 75 developmental milestones for detecting limited intellectual functioning that can be obtained before the age of 4 years. We performed a case-control study with 148 children aged 5–10 years with limited intellectual functioning (IQ 50–69), who were in special education (cases) and a random sample of 300 children aged 5–10 years who were in regular elementary education (controls). Developmental milestones scores were retrieved from Child Healthcare files. We calculated sensitivity, specificity, positive likelihood ratios (LR+) and diagnostic odds ratios (DOR) for limited intellectual functioning. The LR+ determines whether a test result changes the probability that a condition exists. Given the prevalence of intellectual disability (1–3%), we considered that an LR+ > 10 would be clinically useful, as it increases the a priori probability of limited intellectual functioning from 2% to a posteriori probability of at least 17%. Out of 75 assessed milestones, 50 were included in the analysis. We found nine milestones to have a significant adjusted (for socio-economic status and prematurity) DOR > 1 and a significant LR+ > 10 (assessment age in months between brackets): ‘says "dada-baba‴ (9), ‘balances head well while sitting’ (9), ‘sits on buttocks while legs stretched’ (9), ‘babbles while playing’ (12), ‘sits in stable position without support’ (12), ‘walks well alone’ (24), ‘says "sentences" of 3 or more words’ (36), ‘places 3 forms in form-box’ (36) and ‘copies circle’ (48). Sensitivities of these 9 milestones varied from 8–54%, specificities of these 9 milestones varied from 95–100%. Combining these milestones at 9, 12, and 36 months respectively resulted in sensitivities of 27–60% and specificities of 94–99%. These nine developmental milestones have substantial predictive validity for limited intellectual functioning.

## Introduction

Screening instruments to assess developmental delays in children are used worldwide by paediatricians and other professionals in Child Health Care (CHC). They are used for the early identification of a wide range of disabilities and conditions, ranging from severe and rare, e.g., muscular dystrophy [1] or intellectual disability [2], to mild and common, e.g. dyslexia [3] or language delays [4]. Most developmental screening tests have only been validated cross-sectionally against another test, considered to be superior, and not against a “golden standard”. A longitudinal perspective would be more appropriate, and enables to assess the predictive validity of developmental screening tests [5]. The predictive validity refers to the extent a score on a test can predict other measures of the same construct in the future [6].

Next to the fact that the predictive validity is not often studied, when it is studied, a combination of milestones to predict a certain outcome is commonly used [5,7]. The predictive validity of tests during infancy to detect intellectual disabilities that will manifest themselves at a later age has traditionally been poor [8–10].

Developmental milestones are commonly used in practice worldwide [11–13]. In the U.S. almost 90% of paediatricians and family physicians reported to use developmental milestones to screen for developmental delays [11], even though the American Academy of Pediatrics (AAP) recommends the use of standardized developmental screening tools [14]. The consensus seems to be that single milestones will not have sufficient predictive validity. An association between infant motor developmental milestones and intellectual functioning at a later age has been found in some studies [12,15–19]. However, other studies report no association [20,21].

In this study we will use as outcome measure limited intellectual functioning, i.e., an Intelligent Quotient (IQ) between 50 and 69, formerly described as “mild mental retardation” [22].

Early identification of developmental delays in children is important to both professionals and parents, as it may initiate a range of decisions concerning supporting care and (medical) treatment of the child [23–28]. The aim of this study is to determine which developmental milestones have substantial predictive validity for limited intellectual functioning in later life.

## Methods

### Design

We used a case-control design for this study [29]. In Dutch preventive Child Health Care (CHC) the growth and development of all new-born children are routinely measured at regular intervals from birth till the age of 18 years [30]. Results are registered in the personal CHC records.

### Participants

The CHC departments of three Municipal Health Services agreed to participate in the data collection. Cases and controls were recruited from schools situated in the service area of these CHC departments. In CHC, informed consent on anonymous use of filed data is usually given by the parents at their first contact with the CHC department. The Medical Ethical Committee of the Leiden University Medical Centre did not consider the Committee’s approval necessary because only filed data were used in an anonymous way.

Cases were 148 children aged 5–10 years with an IQ ranging from 50 through 69, attending special elementary education (for children with learning disabilities) or special education (for children with a combination of learning disabilities and socio-emotional problems). Children with congenital abnormalities, known to be associated with intellectual disabilities were excluded. Forty-seven percent of the cases were measured with the SON (Snijders-Oomen Non-verbal intelligence test) [31], 29% with the Wechsler Intelligence Scale for Children (WISC) [32], 11% with the Wechsler Preschool and Primary Scale of Intelligence (WPPSI) [33], 10% with the Revised Amsterdam Children’s Intelligence Test (RAKIT) [34] and 1% with other intelligence tests.

Controls were a stratified random sample of 300 children, aged 5–10 years, without a limitation in intellectual functioning, attending regular elementary schools. Stratification was applied by school year and residential area, corresponding to the distribution of these variables in the case group. Children with a recognized intellectual disability or who repeated one of the previous elementary school years were excluded from the control group.

### Data collection

The following data were collected anonymously by one of the authors (EH) from CHC files in 2007: 1) background characteristics, 2) developmental test results at age 0–4 years [35]; and 3) for cases, data on cognitive development (IQ scores), filed in their CHC files as well.

Background characteristics were: age of the child, sex, gestational age, single/ multiple pregnancy, birth weight, Apgar scores 5 minutes after birth, ethnicity, medical history of family members (intellectual disability, epilepsy), age of the mother at delivery, and socioeconomic status (SES) of the parents. Ethnicity was determined according to the definition used by Statistics Netherlands [36]. Children with a Dutch ethnicity have both parents born in the Netherlands. We used the highest educational level completed by father or mother as an indicator for SES. Small for gestational age was defined as birth weight lower than the tenth percentile value for gestational age using Dutch norm tables [37].

### Developmental milestones

In the Netherlands the Dutch Developmental Instrument (DDI), a modification of the Gesell test, is used in preventive CHC to assess development of children from birth to age four years [35,38]. The DDI consists of a set of 75 developmental milestones covering three domains of child development: (1) fine motor activity, adaptive, and personal/social behaviour; (2) communication; and (3) gross motor activity [35]. In a previous study it was shown that application of the Rasch model to the DDI resulted in excellent reliability and satisfactory fit [38]. The DDI is administered by a trained health care professional at 13 routine visits to CHC. These visits are scheduled at the ages 1, 2, 3, 6, 9, 12, 14, 18, 24, 30, 36, 39 and 48 months. Some CHC organizations do not have standard consultation times at the age of 18, 30 and 39 months. Therefore, at these three consultation times many children will have missing data. CHC professionals have a strict set of protocolled activities to monitor a child’s health and development [30,35]. Five to nine specific milestones are administered at each CHC visit. The assessment of these milestones is scheduled at an age at which at least 90% or more of normally developing children will pass [39]. Most of the milestones require assessment by the CHC physician or nurse; some may be registered upon caregivers notice. More detailed information on all milestones and the scheduled assessment age is available in the appendix (S1 Table).

### Statistical analysis

Differences between cases and controls in background characteristics were compared using independent T-tests (for continuous variables) or chi-square tests (for dichotomous variables).

A total of 75 milestones were available for analysis. We included the descriptive statistics only of milestones of which the attendance rate (number of children present at a consultation time divided by the total group) and the assessment rate of a milestone (number of children in which the milestone was assessed divided by the group of present children) were above 70% for both the control group and the case group. For each developmental milestone, sensitivity, specificity, positive likelihood ratio (LR+) and diagnostic odds ratios (DOR) were calculated. The LR+ is calculated as sensitivity/(1-specificity) and determines whether a test result changes the probability that a condition exists. The LR+ presents the probability of a person who has the condition testing positive divided by the probability of a person who does not have the condition testing positive [40]. Considering the low prevalence (1–3%) of intellectual disability [2], an LR+ of 10 or more was considered clinically relevant [41]. For example, not achieving a developmental milestone with an LR+ of 10 would increase the probability of limited intellectual functioning from a pre-test probability of 2% to a post-test probability of 17% (post-test odds = pre-test odds*LR+; pre-test odds = 0.02/(1–0.02) = 0.0204; post-test odds = 0.0204*10 = 0.204; post-test probability 0.204/(0.204+1) = 17%). An LR+ cannot be calculated if specificity is 100% (dividing by zero), which is the case if there are no false positives (control children who did not achieve the milestone). Whenever there were no false positives, an adjusted LR+ was calculated by adding half a point to the number of false and true positives and false and true negatives to prevent a division by 0 [42]. For combinations of developmental milestone with an LR+ of 10 or higher at certain ages, we calculated sensitivity, specificity and positive likelihood ratio (LR+) [43, 44].

SES and gestational age are well known risk factors of limited intellectual functioning [45]. To assess the effect of these two risk factors on developmental milestones with an LR+ > 10 we compared the unadjusted DOR of these milestones with the adjusted DOR of these milestones after controlling for socioeconomic status (SES) and prematurity by using logistic regression. A DOR is a measure of discriminatory performance of a test. This measure combines sensitivity and specificity and is independent of the prevalence [42]. The DOR is a single indicator of test performance. The DOR represents the ratio of the odds of the test being positive if the child has the condition (limited intellectual functioning) divided by the odds of the test being positive if the child does not have the condition. The DOR ratio may also be expressed in terms of the sensitivity and specificity of the test:

DOR = (sensitivity × specificity)/((1 − sensitivity) × (1 − specificity)). For example, a test with a sensitivity of 70% and a specificity of 95% has a DOR of (0.7 x 0.95)/(0.3 x 0.05) = 0.665/0.015 = 44. Tests with a higher DOR have better test properties than tests with a lower DOR. Only milestones with a significant LR+ > 10 as well as a significant adjusted (for socio-economic status and prematurity) DOR > 1 were considered to have substantial predictive validity.

The statistical significance level was set at P<0.05 (two-sided).

## Results

### Background characteristics

All children were born in the Netherlands. Cases did not differ from controls with regard to the mean age at elementary school and the percentage with a Non-Dutch ethnicity (Table 1). Relative to controls, the case group contained more boys, more premature births, more children who were small for gestational age and more children with a less favourable family history (i.e. presence of epilepsy and/or developmental disability). Mean Apgar scores of cases were lower, their mothers were younger and they were more often born in families with lower SES (Table 1). The percentage of children who missed an entire consultation time varied between 0.7% (6 months), and 42.4% (18 months).

**Table 1:** Background characteristics of cases and controls.

		Cases		Controls	
		% or mean (SD)	*n*	% or mean (SD)	*n*
Mean age (years) of child (SD)		8.0 (1,5)	148	7.6 (1.4)	300
Male (vs female) (%)		72.3	148	50.3***	300
Gestational age < 37 (vs ≥ 37) (%)		11.6	146	4.3**	300
Multiple pregnancy (vs single) (%)		6.8	148	3.3	300
Small for gestational age (vs not small for gestational age) (%)		23.4	145	14.0*	300
Mean Apgar score at 5 minutes^a^		9.4 (0.9)	117	9.7 (0.7)**	296
Family medical history: Epilepsy etc.^b^ (vs none) %		16.0	144	6.0*	300
Non-Dutch ethnicity (vs Dutch) %		42.0	143	35.0	300
Mean age (years) mother at delivery		27.8 (5.5)	145	30.3 (4.9)***	299
SES ^c^ (%)			110		277
	Low	6.4		4.0	
	Middle- low	41.8		17.0***	
	Middle- high	37.3		43.7	
	High	14.5		35.4***	

*Notes*.

*p < 0.05

**p < 0.01

***p < 0.001.

^a^ Descriptives are given in original scale. Tests were performed using the log-transformed scale

^b^ Epilepsy and/or intellectual disability

^c^ Highest completed education is taken as an indicator for the SES of the parents.

### Developmental milestones

Out of 75 milestones, 50 had an attendance rate and assessment rate of at least 70% or more for both cases and controls. Of these 50 milestones, 40 had an LR+ below 10 and ten had a significant LR+ above 10 (table in S1 Table). The 10 developmental milestones with an LR+ above 10 are presented in Table 2. The highest LR+, i.e., 46.9, was found for ‘balances head well while sitting’, meaning that not achieving ‘balances head well while sitting’ at the scheduled assessment age of 9 months, increases the probability of limited intellectual functioning from a pre-test probability of 2% to a post-test probability of 48.9%. The specificities of the ten developmental milestones with a significant LR+ above 10 varied between 95% for ‘places 3 forms in form-box’, and 100% for both ‘balances head well while sitting’ and ‘babbles while playing’, meaning that respectively 95% and 100% of children without limited intellectual functioning do achieve these tasks at the scheduled assessment age for these tasks (36 months for ‘places 3 forms in form-box’ and 9 months for both ‘balances head well while sitting’ and ‘babbles while playing’). The sensitivities of the ten developmental milestones with a significant LR+ above 10 varied between 4% for ‘plays with hand in midline’ and 54% for ‘copies a circle’, meaning that respectively 4% and 54% of children with limited intellectual functioning fail on these tasks at the scheduled assessment age for these tasks (6 and 48 months respectively). Six months old was the youngest scheduled assessment age at which a milestone had an LR+ of 10 or more (‘plays with hands in midline’). Combining milestones at 9, 12, and 36 months respectively resulted in sensitivities of 27–60% and specificities of 94–99%.

**Table 2:** Developmental domain, age, specificity, sensitivity and LR+ of developmental milestones with an LR+ above 10^a^.

Milestone	Develop-mental domain^b^	Scheduled assessment age in months	Assessment age in months (mean ± standard deviation)			Specificity (95% CI^c^) (%)	Sensitivity (95% CI) (%)	LR+ (95% CI^d^)
			Controls	Cases				
1. Plays with hands in midline	F	6	6.6 ± 0.6	6.7 ± 0.6		99.6 (98.0–99.9)	4.1 (1.7–9.2)	11.6 (1.4–98.1)
2. Says "dada-baba" or "gaga"^e^	C	9	9.7 ± 0.5	9.9 ± 0.5	*	99.6 (98.0–99.9)	8.7 (4.9–15.0)	24.5 (3.2–188.0)
3. Balances head well while sitting	G	9	9.7 ± 0.5	9.9 ± 0.5	*	100.0 (98.6–100.0)	8.1 (4.4–14.2)	46.9 (2.8–793.7)^c^
4. Sits on buttocks while legs stretched	G	9	9.7 ± 0.5	9.9 ± 0.5	*	98.2 (95.8–99.2)	23.6 (16.9–31.8)	12.8 (5.1–32.3)
**At least one negative score on the above milestones at 9 months**	C, G	9	8.9 ± 0.5	9.1 (0.5)	*	97.7 (95.1–99.0)	28.3 (21.0–37.0)	12.5 (5.4–29.0)
5. Babbles while playing^e^	C	12	12.7 ± 0.6	12.7 ± 0.6		100.0 (98.1–100.0)	8.2 (4.0–16.0)	34.5 (2.0–598.0)^c^
6. Sits in stable position, without support	G	12	12.7 ± 0.6	12.7 ± 0.6		99.1 (96.9–99.8)	17.6 (11.1–26.7)	20.6 (4.8–87.7)
**At least one negative score on the above milestones at 12 months**	C, G	12	11.7 ± 0.5	11.7 ± 0.5		98.9 (96.2–99.7)	27.4 (18.5–38.6)	25.5 (6.1–106.3)
7. Walks well alone	G	24	24.3 ± 1.0	24.7 ± 1.5	*	98.9 (96.8–99.6)	21.0 (14.7–29.2)	19.0 (5.9–61.9)
8. Says "sentences" of 3 or more words^e^	C	36	36.4 ± 1.2	36.6 ± 1.8		98.6 (96.3–99.4)	36.0 (27.3–45.8)	24.9 (9.1–68.3)
9. Places 3 forms in form-box	F	36	36.4 ± 1.2	36.6 ± 1.8		95.3 (92.1–97.2)	50.0 (40.0–60.0)	10.5 (6.0–18.6)
**At least one negative score on the above milestones at 36 months**	C, F	36	36.5 ± 1.1	36.8 ± 1.5		94.0 (90.1–96.3)	60.2 (49.5–70.1)	10.02 (6.0–16.6)
10. Copies a circle	F	48	46.6 ± 1.7	47.4 ± 2.2	*	96.1 (93.0–97.9)	53.6 (43.0–63.8)	13.8 (7.3–26.2)

Notes.

*p < 0.001.

^a^ Items are only presented when both presence at the consultation time and response on an item when present at the consultation time was at least 70% for both cases and controls.

^b^C = communication, F = fine motor activity, adaptive and personal/social behavior, G = gross motor activity

^c^Confidence intervals for sensitivity and specificity are calculated with the Wilson score method [43]

^d^Confidence intervals for positive likelihood ratios are produced with the method described by Simel and colleagues [44]

^e^Information about milestone may be registered upon caregivers notice.

We adjusted for SES and prematurity in the models for the ten milestones with an LR+ above 10 (Table 3). Only the adjusted DOR of the milestone “plays with hands in midline” was not statistically significant anymore. Before adjustment the DOR’s of the other nine milestones varied from 16.5 to 38.4. After adjustment the DOR’s of these nine milestones varied from 16.2 to 72.9. In other words, the adjusted DOR’s of these nine milestones remained high and statistically significant. Meaning that these nine milestones have substantial predictive validity for limited intellectual functioning.

**Table 3:** Diagnostic odds ratios (DOR) for limited intellectual functioning of developmental milestones with an LR+ above 10.

Milestone	Crude DOR (95% CI)	Adjusted DOR^a^ (95% CI)
Plays with hands in midline	12.0 (1.4–104.1)	4.6 (0.4–48.1)
Says "dada-baba"or "gaga"	26.8 (3.4–209.8)	30.7 (3.5–265.4)
Balances head well while sitting	24.3 (3.1–192.0)	18.3 (2.1–160.3)
Sits on buttocks while legs stretched	16.5 (6.2–43.8)	23.7 (7.7–73.3)
Babbles while playing	17.6 (2.1–145.3)	17.8 (1.9–166.5)
Sits in stable position. without support	24.7 (5.6–110.1)	24.5 (4.9–121.2)
Walks well alone	23.8 (7.0–80.8)	36.9 (9.8–138.6)
Says "sentences" of 3 or more words	38.4 (13.2–111.7)	72.9 (16.2–327.6)
Places 3 forms in form-box	20.1 (10.1–40.1)	16.2 (7.2–36.4)
Copies a circle	28.6 (13.3–61.4)	17.9 (7.8–41.2)

^a^Adjusted for prematurity and SES of parents

## Discussion

We examined the predictive validity of single developmental milestones for detecting a limitation in intellectual functioning. Our study shows that the majority of developmental milestones do not have substantial predictive validity (LR+ below 10), while only nine developmental milestones have substantial predictive validity (significant LR+ > 10 and a significant DOR > 1) for detecting children with limited intellectual functioning.

As expected, cases and controls differed significantly on several background characteristics. Cases were more often of male sex, premature, small for gestational age and had more often a low Apgar score at 5 minutes, less favourable family medical history, young age of the mother at delivery and low SES. These characteristics are known to be risk factors for intellectual disabilities [45,46].

The developmental milestones with an LR+ >10 came from all three domains of child development, i.e. 1) fine motor activity, adaptive, and personal/social behaviour, 2) communication and 3) gross motor activity. This is in agreement with findings that children with a low IQ usually have difficulties with communication tasks as well as impaired motor skills [47]. Although in this study we did not investigate the interrelation between milestones, recent research suggests that milestones from different domains might also be interrelated. For example, in a prospective cohort study the effects of early motor development on adult intelligence was shown to be associated with and partly mediated by later language acquisition [48]. This might imply that earlier attainment of motor development skills such as walking, increases opportunities for exploring the environment and interactions with caregivers, which may stimulate cognitive and language development [48]. Another reason for the significant association between motor development and intelligence includes shared genetic aetiology and shared environmental causes [48].

We found that gross motor milestones ‘Balances head well while sitting’, ‘Sits on buttocks while legs stretched’, ‘Sits in stable position without support’, and ‘Walks well alone’, have substantial predictive validity for detecting a limitation in intellectual functioning. This finding is in contrast with two other studies reporting no associations between motor milestones and later intellectual functioning [20,21]. In these studies, the authors question the stability of motor development during early childhood and argue that milestone attainment would have low prognostic value for later intellectual functioning. However, there are several other studies that corroborate our finding that there is an association between infant motor milestones and intellectual functioning at a later age [12,15–19].

Three communication milestones have substantial predictive validity for detecting a limitation in intellectual functioning, i.e., ‘Says "dada-baba"or "gaga‴, ‘Babbles while playing’, ‘Says "sentences" of 3 or more words’. That communication milestones are important for intellectual functioning in later life was also confirmed in an earlier study in which the milestone of speech attainment was found to be associated with IQ at the age of 8 years [16].

The fine motor skills milestones ‘Plays with hands in midline’, ‘Places 3 forms in form-box’ and ‘Copies a circle’ have substantial predictive validity for intellectual functioning. The relation between these fine motor skills milestones and intellectual functioning was, to our knowledge, not investigated in earlier studies. However, children with limited intellectual functioning are known to have more deficits in fine motor skills, as compared to children with normal intellectual functioning [49].

In a study from 1982, the age of milestone attainment was found to be related to later intellectual functioning [50]. However, when background characteristics were included in the model, these variables overshadowed the developmental milestones as predictors of intellectual functioning. In contrast, in our study, we show that some developmental milestones remain substantially predictive of intellectual functioning, even when SES and prematurity are included in the model.

The specificities of the developmental milestones in our study were quite high. The DDI was intentionally constructed in a way that the assessment time of the milestones is scheduled after the age at which at least 90% of children are expected to achieve the developmental milestone, to limit the number of false positives [39]. Therefore, many milestones have a substantially higher specificity than 90%. This may explain why the sensitivities of the developmental milestones were in general not high at the assessment age. However, combining the milestones at 36 months resulted in a sensitivity of 60% and a specificity of 94%, which implies that 6 out of 10 children with a low IQ can be found at 36 months, which gives parents time to find schools that fit the need of their child.

The practical relevance of our findings is that milestones with an LR+ > 10 are important to detect limited intellectual functioning. Not achieving such a developmental milestone at the assessment age should alarm the professional, as the chance that the child will have limited intellectual functioning at a later age is increased from 2% (the estimated prevalence or a priori probability of limited intellectual functioning) to an a posteriori probability of at least 17% (see method section). However, due to low sensitivity, achieving a developmental milestone at the assessment age does not rule out limited intellectual functioning. We expect that a model that includes a combination of relevant developmental milestones and demographic variables will predict better whether a child has limited intellectual functioning. Therefore, further research is necessary to determine if a more comprehensive model can increase sensitivity while retaining a high specificity.

One of the strengths of our study is its prospective nature. All developmental milestones were assessed in preventive CHC before the children were known to have limited intellectual functioning. Another strength was that limited intellectual functioning was measured at an age at which IQ can be assessed reliably [31–34]. Our study can be considered as an effectiveness study rather than an efficacy study, because the milestones were assessed during regular practice and not during a well-controlled study. This strengthens the generalizability of our results.

We used a case-control design for our study instead of a cohort study because it is more efficient and less costly. A cohort study would have forced us to follow more than 7500 children for several years to include the same amount of cases, as the prevalence of an intellectual disability is between 1–3%.

A limitation of our study is that one cannot directly estimate what the positive predictive value is, since we oversampled cases. However, in an indirect way this is possible. With the LR+ and an estimate of the prevalence of limited intellectual functioning (about 1–3%), estimates of the positive predictive value can be made (see methods section for further clarification).

Another limitation of our study is that a few cases may have wrongfully been included in the control group. In the Netherlands, the usual policy is to keep children with developmental and/or intellectual problems in regular education as long as possible. These children usually repeat a class. For that reason we have excluded children who repeated a class from the study. If nevertheless a few cases were included in the control group this would mean that specificity will be (slightly) underestimated in our study. Similarly, it is possible that children who failed developmental milestones received early intervention and, as a consequence did not develop limited intellectual functioning [7]. Although there are wide varieties in the aetiology of intellectual functioning, including genetic factors which cannot be prevented, increasing parent-sensitive responsiveness has been found to improve child outcomes in some children with an intellectual or developmental delay [51], for example in verbal IQ [52]. If it is the case that some children received early intervention, these children could have been included in the control group, which would also lead to an underestimation of specificity. However, the number of cases with limited intellectual functioning is small and the number of cases that has received early intervention and as a consequence did not develop limited intellectual functioning will be even smaller. Therefore, we expect that this bias on specificity will be negligible.

We conclude that the majority of developmental milestones do not have substantial predictive validity for detecting limited intellectual functioning. However, some milestones do have substantial predictive validity and professionals in CHC should be alert when a child is unable to achieve one of these developmental milestones at the assessment age.

## Supporting information

S1 Table Age, number of false and true positives and negative, specificity, sensitivity and LR+ of developmental milestones of the DDI. (PDF)
