# Supplementary material for: Predictive validity of developmental milestones for detecting limited intellectual functioning
Source: PLoS One. 2019 Mar 28;14(3):e0214475. doi: 10.1371/journal.pone.0214475 (PMC6438572; doi:10.1371/journal.pone.0214475)
Supplement: S1 Table — (PDF) [file pone.0214475.s001.pdf]

S1 Table. Age, number of false and true positives and negative, specificity, sensitivity and LR+ of developmental milestones of the DDI.

N°	Developmental milestone	Scheduled assessment age in months	Assessment age in months (mean ± standard deviation)		False positives	True negatives	True positives	False negatives	Specificity (CI[a]) (%)	Sensitivity (CI) (%)	LR+ (CI[b])
			**Controls**	**Cases**							
1	Reacts when spoken to	1	1.1 ± 0.2	1.1 ± 0.3	5	271	6	110	98.2 (95.8 - 99.2)	5.2 (2.4 - 10.8)	2.9 (0.9 - 9.2)
2	Eyes fixate	1	1.1 ± 0.2	1.1 ± 0.3	5	268	5	114	98.2 (95.8 - 99.2)	4.2 (1.8 - 9.5)	2.3 (0.7 - 7.8)
3	Moves arms equally well	1	1.1 ± 0.2	1.1 ± 0.3	0	278	1	114	100.0 (98.6 - 100.0)	0.9 (0.2 - 4.8)	7.2 (0.3 - 175.8)[c]
4	Moves legs equally well	1	1.1 ± 0.2	1.1 ± 0.3	0	278	0	114	100.0 (98.6 - 100.0)	0.0 (0.0 - 3.3)	2.4 (0.0 - 121.5)[c]
5	Lifts chin off table for a moment	1	1.1 ± 0.2	1.1 ± 0.3	16	261	7	108	94.2 (90.8 - 96.4)	6.1 (3.0 - 12.0)	1.1 (0.4 - 2.5)
6	Stays suspended when lifted under armpits	1	1.1 ± 0.2	1.1 ± 0.3	2	268	1	97	99.3 (97.3 - 99.8)	1.0 (0.2 - 5.6)	1.4 (0.1 - 15.0)
7	Reactions if pulled to sitting	1	1.1 ± 0.2	1.1 ± 0.3	2	240	7	91	99.2 (97.0 - 99.8)	7.1 (3.5 - 14.0)	8.6 (1.8 - 40.9)
8	Smiles in response	2	2.1 ± 0.4	2.1 ± 0.3	6	178	6	61	96.7 (93.1 - 98.5)	9.0 (4.2 - 18.2)	2.7 (0.9 - 8.2)
9	Follows with eyes and head 30° < 0° > 30°	2	2.1 ± 0.4	2.1 ± 0.3	19	143	16	44	88.3 (82.4 - 92.4)	26.7 (17.1 - 39.0)	2.3 (1.3 - 4.1)
10	Watches own hands	3	3.4 ± 0.5	3.4 ± 0.4	14	271	12	104	95.1 (91.9 - 97.1)	10.3 (6.0 - 17.2)	2.1 (1.0 - 4.4)
11	Vocalizes in response	3	3.4 ± 0.5	3.4 ± 0.4	3	283	1	123	99.0 (97.0 - 99.6)	0.8 (0.1 - 4.4)	0.8 (0.1 - 7.3)
12	Hands occasionally open	3	3.4 ± 0.5	3.4 ± 0.4	2	281	2	113	99.3 (97.5 - 99.8)	1.7 (0.5 - 6.1)	2.5 (0.4 - 17.3)
13	Lifts head to 45° in prone position	3	3.4 ± 0.5	3.4 ± 0.4	45	239	29	93	84.2 (79.5 - 87.9)	23.8 (17.1 - 32.1)	1.5 (1.0 - 2.3)
14	Produces varying sounds	6	6.6 ± 0.6	6.7 ± 0.6	3	275	1	117	98.9 (96.9 - 99.6)	0.8 (0.1 - 4.6)	0.8 (0.1 - 7.5)
15	Responds to name	6	6.6 ± 0.6	6.7 ± 0.6	18	226	21	102	92.6 (88.6 - 95.3)	17.1 (11.4 - 24.7)	2.3 (1.3 - 4.2)
16	Plays with hands in midline	6	6.6 ± 0.6	6.7 ± 0.6	1	284	5	118	99.6 (98.0 - 99.9)	4.1 (1.7 - 9.2)	11.6 (1.4 - 98.1)
17	Supine position: grasps object within reach	6	6.6 ± 0.6	6.7 ± 0.6	7	273	12	115	97.5 (94.9 - 98.8)	9.4 (5.5 - 15.8)	3.8 (1.5 - 9.4)
18	Looks around to side with angle face-table 90°	6	6.6 ± 0.6	6.7 ± 0.6	11	272	16	109	96.1 (93.2 - 97.8)	12.8 (8.0 - 19.8)	3.3 (1.6 - 6.9)
19	Flexes or stomps legs while being swung	6	6.6 ± 0.6	6.7 ± 0.6	8	267	7	115	97.1 (94.4 - 98.5)	5.7 (2.8 - 11.4)	2.0 (0.7 - 5.3)
20	Says "dada-baba" or "gaga"	9	9.7 ± 0.5	9.9 ± 0.5	1	280	11	115	99.6 (98.0 - 99.9)	8.7 (4.9 - 15.0)	24.5 (3.2 -

S1 Table. Age, number of false and true positives and negative, specificity, sensitivity and LR+ of developmental milestones of the DDI.

N°	Developmental milestone	Scheduled assessment age in months	Assessment age in months (mean ± standard deviation)		False positives	True negatives	True positives	False negatives	Specificity (CI[a]) (%)	Sensitivity (CI) (%)	LR+ (CI[b])
											188.0)
21	Rolls over, back and forth	9	9.7 ± 0.5	9.9 ± 0.5	22	258	23	96	92.1 (88.4 - 94.8)	19.3 (13.2 - 27.3)	2.5 (1.4 - 4.2)
22	Passes cube from hand to hand	9	9.7 ± 0.5	9.9 ± 0.5	7	265	10	106	97.4 (94.8 - 98.7)	8.6 (4.7 - 15.1)	3.3 (1.3 - 8.6)
23	Holds cube, grasps another one with other hand	9	9.7 ± 0.5	9.9 ± 0.5	8	265	17	101	97.1 (94.3 - 98.5)	14.4 (9.2 - 21.9)	4.9 (2.2 - 11.1)
24	Plays with both feet	9	9.7 ± 0.5	9.9 ± 0.5	4	268	7	112	98.5 (96.3 - 99.4)	5.9 (2.9 - 11.6)	4.0 (1.2 - 13.4)
25	Balances head well while sitting	9	9.7 ± 0.5	9.9 ± 0.5	0	278	10	114	100.0 (98.6 - 100.0)	8.1 (4.4 - 14.2)	46.9 (2.8 - 793.7)[c]
26	Sits on buttocks while legs stretched	9	9.7 ± 0.5	9.9 ± 0.5	5	267	29	94	98.2 (95.8 - 99.2)	23.6 (16.9 - 31.8)	12.8 (5.1 - 32.3)
27	Crawls forward, abdomen on the floor	12	12.7 ± 0.6	12.7 ± 0.6	21	171	31	62	89.1 (83.9 - 92.7)	33.3 (24.6 - 43.4)	3.0 (1.9 - 5.0)
28	Pulls up to standing position	12	12.7 ± 0.6	12.7 ± 0.6	27	199	40	56	88.1 (83.2 - 91.7)	41.7 (32.3 - 51.7)	3.5 (2.3 - 5.3)
29	Babbles while playing	12	12.7 ± 0.6	12.7 ± 0.6	0	197	7	78	100.0 (98.1 - 100.0)	8.2 (4.0 - 16.0)	34.5 (2.0 - 598.0)[c]
30	Waves "bye-bye"	12	12.7 ± 0.6	12.7 ± 0.6	12	173	22	55	93.5 (89.0 - 96.3)	28.6 (19.7 - 39.5)	4.4 (2.3 - 8.4)
31	Picks up pellet between thumb and index finger	12	12.7 ± 0.6	12.7 ± 0.6	8	174	20	48	95.6 (91.6 - 97.8)	29.4 (19.9 - 41.1)	6.7 (3.1 - 14.5)
32	Sits in stable position, without support	12	12.7 ± 0.6	12.7 ± 0.6	2	232	16	75	99.1 (96.9 - 99.8)	17.6 (11.1 - 26.7)	20.6 (4.8 - 87.7)
33	Reacts to a verbal request	12	12.7 ± 0.6	12.7 ± 0.6	5	158	10	54	96.9 (93.0 - 98.7)	15.6 (8.7 - 26.4)	5.1 (1.8 - 14.3)
34	Puts cube in and out of a box	14	14.5 ± 0.9	14.4 ± 0.9	6	267	19	87	97.8 (95.3 - 99.0)	17.9 (11.8 - 26.3)	8.2 (3.3 - 19.9)
35	Plays "give and take"	14	14.5 ± 0.9	14.4 ± 0.9	8	257	18	91	97.0 (94.2 - 98.5)	16.5 (10.7 - 24.6)	5.5 (2.5 - 12.2)
36	Understands a few daily-used sentences	14	14.5 ± 0.9	14.4 ± 0.9	7	265	19	99	97.4 (94.8 - 98.7)	16.1 (10.6 - 23.8)	6.3 (2.7 - 14.5)
37	Says 2 "sound-words" with comprehension	14	14.5 ± 0.9	14.4 ± 0.9	33	233	41	81	87.6 (83.1 - 91.0)	33.6 (25.8 - 42.4)	2.7 (1.8 - 4.1)
38	Crawls, abdomen off the floor	14	14.5 ± 0.9	14.4 ± 0.9	12	266	23	97	95.7 (92.6 - 97.5)	19.2 (13.1 - 27.1)	4.4 (2.3 - 8.6)
39	Walks along	14	14.5 ± 0.9	14.4 ± 0.9	7	268	20	102	97.5 (94.8 - 98.8)	16.4 (10.9 - 24.0)	6.4 (2.8 - 14.8)

S1 Table. Age, number of false and true positives and negative, specificity, sensitivity and LR+ of developmental milestones of the DDI.

N°	Developmental milestone	Scheduled assessment age in months	Assessment age in months (mean ± standard deviation)		False positives	True negatives	True positives	False negatives	Specificity (CI[a]) (%)	Sensitivity (CI) (%)	LR+ (CI[b])
40	Builds tower of 2 cubes	18	17.7 ± 2.2	18.1 ± 1.6	15	33	22	25	68.8 (54.7 - 80.1)	46.8 (33.3 - 60.8)	1.5 (0.9 - 2.5)
41	Explores environment	18	17.7 ± 2.2	18.1 ± 1.6	1	56	3	58	98.2 (90.7 - 99.7)	4.9 (1.7 - 13.5)	2.8 (0.3 - 26.2)
42	Says 3 "words"	18	17.7 ± 2.2	18.1 ± 1.6	4	71	18	41	94.7 (87.1 - 97.9)	30.5 (20.3 - 43.1)	5.7 (2.0 - 16.0)
43	Understands "play" orders	18	17.7 ± 2.2	18.1 ± 1.6	0	53	10	46	100.0 (93.2 - 100.0)	17.9 (10.0 - 29.8)	19.9 (1.2 - 331.3)[c]
44	Walks alone	18	17.7 ± 2.2	18.1 ± 1.6	3	148	22	52	98.0 (94.3 - 99.3)	29.7 (20.5 - 40.9)	15.0 (4.6 - 48.4)
45	Throws ball without falling down	18	17.7 ± 2.2	18.1 ± 1.6	4	35	20	25	89.7 (76.4 - 95.9)	44.4 (30.9 - 58.8)	4.3 (1.6 - 11.6)
46	Imitates others	24	24.3 ± 1.0	24.7 ± 1.5	4	263	9	103	98.5 (96.2 - 99.4)	8.0 (4.3 - 14.6)	5.4 (1.7 - 17.1)
47	Builds tower of 3 cubes	24	24.3 ± 1.0	24.7 ± 1.5	15	234	40	69	94.0 (90.3 - 96.3)	36.7 (28.2 - 46.1)	6.1 (3.5 - 10.5)
48	Says "sentences" of 2 words	24	24.3 ± 1.0	24.7 ± 1.5	31	240	79	46	88.6 (84.2 - 91.8)	63.2 (54.5 - 71.1)	5.5 (3.9 - 7.9)
49	Points at 6 parts of a body of a doll	24	24.3 ± 1.0	24.7 ± 1.5	18	238	55	48	93.0 (89.2 - 95.5)	53.4 (43.8 - 62.7)	7.6 (4.7 - 12.3)
50	Squats or bends to pick up things	24	24.3 ± 1.0	24.7 ± 1.5	6	259	18	100	97.7 (95.1 - 99.0)	15.3 (9.9 - 22.8)	6.7 (2.7 - 16.5)
51	Walks well alone	24	24.3 ± 1.0	24.7 ± 1.5	3	269	25	94	98.9 (96.8 - 99.6)	21.0 (14.7 - 29.2)	19.0 (5.9 - 61.9)
52	Undresses himself	30	26.9 ± 3.3	29.1 ± 2.9	9	69	12	36	88.5 (79.5 - 93.8)	25.0 (14.9 - 38.8)	2.2 (1.0 - 4.8)
53	Builds tower of 6 cubes	30	26.9 ± 3.3	29.1 ± 2.9	3	169	14	42	98.3 (95.0 - 99.4)	25.0 (15.5 - 37.7)	14.3 (4.3 - 48.1)
54	Places round form in form-box	30	26.9 ± 3.3	29.1 ± 2.9	3	120	7	56	97.6 (93.1 - 99.2)	11.1 (5.5 - 21.2)	4.6 (1.2 - 17.0)
55	Refers to self using "me" or "I"	30	26.9 ± 3.3	29.1 ± 2.9	4	107	34	35	96.4 (91.1 - 98.6)	49.3 (37.8 - 60.8)	13.7 (5.1 - 36.9)
56	Points at 5 pictures in the book	30	26.9 ± 3.3	29.1 ± 2.9	2	99	32	34	98.0 (93.1 - 99.5)	48.5 (36.8 - 60.3)	24.5 (6.1 - 98.7)
57	Kicks ball	30	26.9 ± 3.3	29.1 ± 2.9	6	91	9	48	93.8 (87.2 - 97.1)	15.8 (8.5 - 27.4)	2.6 (1.0 - 6.8)
58	Can rotate fluently in sitting position	30	26.9 ± 3.3	29.1 ± 2.9	1	69	10	31	98.6 (92.3 - 99.7)	24.4 (13.8 - 39.3)	17.1 (2.3 - 128.6)
59	Says "sentences" of 3 or more words	36	36.4 ± 1.2	36.6 ± 1.8	4	273	36	64	98.6 (96.3 - 99.4)	36.0 (27.3 - 45.8)	24.9 (9.1 - 68.3)
60	Speech is understood by acquaintances	36	36.4 ± 1.2	36.6 ± 1.8	14	255	41	48	94.8 (91.5 - 96.9)	46.1 (36.1 - 56.4)	8.9 (5.1 - 15.5)

S1 Table. Age, number of false and true positives and negative, specificity, sensitivity and LR+ of developmental milestones of the DDI.

N°	Developmental milestone	Scheduled assessment age in months	Assessment age in months (mean ± standard deviation)		False positives	True negatives	True positives	False negatives	Specificity (CI[a]) (%)	Sensitivity (CI) (%)	LR+ (CI[b])
61	Rides (tri)cycle	36	36.4 ± 1.2	36.6 ± 1.8	22	240	45	43	91.6 (87.6 - 94.4)	51.1 (40.9 - 61.3)	6.1 (3.9 - 9.5)
62	Imitates building a truck	36	36.4 ± 1.2	36.6 ± 1.8	31	225	59	27	87.9 (83.3 - 91.3)	68.6 (58.2 - 77.4)	5.7 (4.0 - 8.1)
63	Places 3 forms in form-box	36	36.4 ± 1.2	36.6 ± 1.8	13	261	46	46	95.3 (92.1 - 97.2)	50.0 (40.0 - 60.0)	10.5 (6.0 - 18.6)
64	Imitates drawing a vertical line	36	36.4 ± 1.2	36.6 ± 1.8	18	223	42	44	92.5 (88.5 - 95.2)	48.8 (38.6 - 59.2)	6.5 (4.0 - 10.7)
65	Walks smoothly	36	36.4 ± 1.2	36.6 ± 1.8	2	263	17	63	99.2 (97.3 - 99.8)	21.3 (13.7 - 31.4)	28.2 (6.6 - 119.3)
66	Puts on own garment	39	39.2 ± 4.0	40.1 ± 3.8	8	63	29	16	88.7 (79.3 - 94.2)	64.4 (49.8 - 76.8)	5.7 (2.9 - 11.4)
67	Talks spontaneously about events at home/playgroup	39	39.2 ± 4.0	40.1 ± 3.8	5	71	24	10	93.4 (85.5 - 97.2)	70.6 (53.8 - 83.2)	10.7 (4.5 - 25.7)
68	Asks questions about "who", "what", "where" and "how"	39	39.2 ± 4.0	40.1 ± 3.8	5	67	30	12	93.1 (84.8 - 97.0)	71.4 (56.4 - 82.8)	10.3 (4.3 - 24.5)
69	Imitates building a bridge	39	39.2 ± 4.0	40.1 ± 3.8	7	57	45	7	89.1 (79.1 - 94.6)	86.5 (74.7 - 93.3)	7.9 (3.9 - 16.0)
70	Places 4 forms in form-box	39	39.2 ± 4.0	40.1 ± 3.8	7	131	39	16	94.9 (89.9 - 97.5)	70.9 (57.9 - 81.2)	14.0 (6.7 - 29.3)
71	Jumps with both feet simultaneously	39	39.2 ± 4.0	40.1 ± 3.8	5	51	11	14	91.1 (80.7 - 96.1)	44.0 (26.7 - 62.9)	4.9 (1.9 - 12.7)
72	Asks questions about "how much", "when", and "why"	48	46.6 ± 1.7	47.4 ± 2.2	5	250	32	46	98.0 (95.5 - 99.2)	41.0 (30.8 - 52.1)	20.9 (8.4 - 51.9)
73	Holds pencil with fingers	48	46.6 ± 1.7	47.4 ± 2.2	19	246	37	62	92.8 (89.1 - 95.4)	37.4 (28.5 - 47.2)	5.2 (3.2 - 8.6)
74	Copies a circle	48	46.6 ± 1.7	47.4 ± 2.2	10	248	45	39	96.1 (93.0 - 97.9)	53.6 (43.0 - 63.8)	13.8 (7.3 - 26.2)
75	Speech is easily understood by examiner	48	46.6 ± 1.7	47.4 ± 2.2	25	230	46	38	90.2 (85.9 - 93.3)	54.8 (44.1 - 65.0)	5.6 (3.7 - 8.5)

Notes. [a]Confidence intervals for sensitivity and specificity are calculated with the Wilson score method(44); [b]Confidence intervals for positive likelihood ratios are produced with the method described by Simel and colleagues (45), [c]Corrected LR+.
